# *trans*-Diaqua­bis­[2-(2-pyrid­yl)acetato-κ^2^ *N*,*O*]nickel(II)

**DOI:** 10.1107/S1600536810030904

**Published:** 2010-08-11

**Authors:** Hong Zhou, Lei Zhao, Rong Huang, Hao-Liang Li

**Affiliations:** a College of Basic Science and Information Engineering, Yunnan Agricultural University, Kunming 650201, People’s Republic of China

## Abstract

In the centrosymmetric title complex, [Ni(C_7_H_6_NO_2_)_2_(H_2_O)_2_], the Ni^II ^atom, located on an inversion center, is six-coordinated in a distorted octa­hedral geometry defined by two N and four O atoms from the two chelating 2-(2-pyrid­yl)acetate ligands and two aqua ligands. The mol­ecules form a three-dimensional framework by O—H⋯O hydrogen bonds and aromatic π–π stacking inter­actions, with a centroid–centroid distance of 3.506 (3) Å.

## Related literature

For similar structures, see: Faure & Loiseleur (1972 ▶, 1975 ▶).
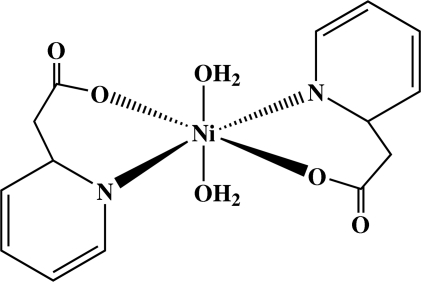


## Experimental

### 

#### Crystal data

[Ni(C_7_H_6_NO_2_)_2_(H_2_O)_2_]
*M* *_r_* = 367.00
Monoclinic, 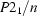

*a* = 8.3346 (12) Å
*b* = 7.100 (1) Å
*c* = 12.1023 (18) Å
β = 102.977 (2)°
*V* = 697.87 (17) Å^3^
*Z* = 2
Mo *K*α radiation
μ = 1.43 mm^−1^
*T* = 293 K
0.22 × 0.15 × 0.11 mm

#### Data collection

Bruker APEXII 1K CCD area-detector diffractometer
Absorption correction: multi-scan (*SADABS*; Bruker, 2004 ▶) *T*_min_ = 0.774, *T*_max_ = 0.855
4515 measured reflections
1627 independent reflections
1471 reflections with *I* > 2σ(*I*)
*R*_int_ = 0.017

#### Refinement

*R*[*F* ^2^ > 2σ(*F* ^2^)] = 0.023
*wR*(*F* ^2^) = 0.067
*S* = 1.00
1627 reflections
114 parameters
H atoms treated by a mixture of independent and constrained refinement
Δρ_max_ = 0.34 e Å^−3^
Δρ_min_ = −0.20 e Å^−3^

### 

Data collection: *APEX2* (Bruker, 2004 ▶); cell refinement: *SAINT* (Bruker, 2004 ▶); data reduction: *SAINT*; program(s) used to solve structure: *SHELXS97* (Sheldrick, 2008 ▶); program(s) used to refine structure: *SHELXL97* (Sheldrick, 2008 ▶); molecular graphics: *SHELXTL* (Sheldrick, 2008 ▶); software used to prepare material for publication: *SHELXTL*.

## Supplementary Material

Crystal structure: contains datablocks I, global. DOI: 10.1107/S1600536810030904/gk2294sup1.cif

Structure factors: contains datablocks I. DOI: 10.1107/S1600536810030904/gk2294Isup2.hkl

Additional supplementary materials:  crystallographic information; 3D view; checkCIF report

## Figures and Tables

**Table:** 

Ni1—O2	2.0397 (10)
Ni1—N1	2.0789 (13)
Ni1—O1*W*	2.1228 (11)

**Table:** 

O2^i^—Ni1—N1^i^	88.90 (4)
O2^i^—Ni1—N1	91.10 (4)
N1^i^—Ni1—N1	180
O2^i^—Ni1—O1*W*	94.53 (4)
N1—Ni1—O1*W*	91.70 (5)
O2^i^—Ni1—O1*W*^i^	85.47 (5)
N1—Ni1—O1*W*^i^	88.30 (5)
O1*W*—Ni1—O1*W*^i^	180

Symmetry code: (i) 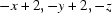.

**Table 2:** Hydrogen-bond geometry (Å, °)

*D*—H⋯*A*	*D*—H	H⋯*A*	*D*⋯*A*	*D*—H⋯*A*
O1*W*—H1*WA*⋯O1^ii^	0.84 (2)	1.97 (2)	2.8035 (17)	169.7 (19)
O1*W*—H1*WB*⋯O1^iii^	0.87 (3)	1.93 (3)	2.7936 (17)	169 (2)

Symmetry codes: (ii) 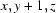; (iii) 
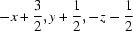
.

**Fig. 1.**
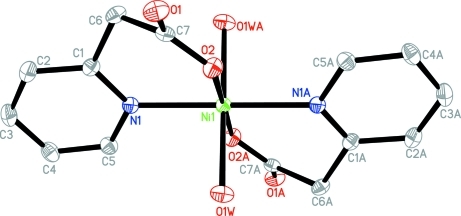
The molecular structure of the title complex with the atom-numbering scheme. Displacement ellipsoids are drawn at the 30% probability level. H-atoms have been omitted. Symmetry code for the atoms with the A label: 2-x, 2-y, -z.

**Fig. 2.**
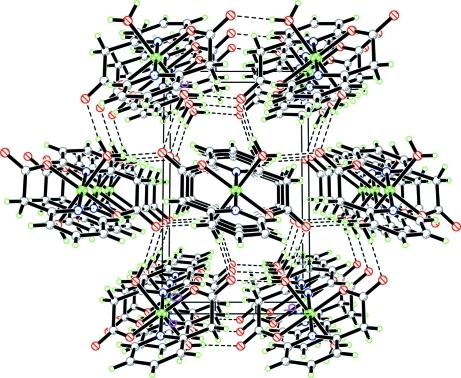
Crystal packing diagram with hydrogen bonds shown by dashed lines.

## supplementary crystallographic information

### Comment

(2-Pyridinyl)acetic acid is a common ligand. Here we report the synthesis of [Ni(C_5_H_4_NCH_2_CO_2_)_2_(H_2_O)_2_], in which the Ni(II) ion coordination environment is the same as in [Zn(C_5_H_4_NCH_2_CO_2_)_2_(H_2_O)_2_] reported earlier (Faure & Loiseleur,1972). The Zn and Ni complexes show a high degree of isostructurality.

As shown in Fig. 1, the Ni(II) coordination geometry can be considered as a distorted octahedral with N_2_O_4 _donor set. Due to a special position of Ni(II), the complex molecule is centrosymmetric. The atoms N1, O2, N1^i^, O2^i^ (symmetry code i: -*x* + 2, -*y* + 2, -*z*) from the (2-pyridinyl)acetate ligand are located in the equatorial plane, while O1W and O1W^i^ are in the axial positions. In the title complex the (2-pyridinyl)acetate anion acts as a chelating bidentate ligand.

Two kinds of intermolecular O—H···O hydrogen bonds (Table 1) were found which link the neighboring molecules into two dimensional layers parallel to the *ab* plane. The two-dimensional layers are assembled *via* weak aromatic π-π stacking interactions into three-dimensional network with a centroid-to-centroid distance of 3.506 (3) Å.

### Experimental

All the chemicals and solvents used for the syntheses were of reagent grade and used without further purification. Ni(CH_3_COO)_2_.4H_2_O (24.88 mg, 0.1 mmol) was dissolved in 5 ml of H_2_O, while (2-pyridinyl)acetic acid (27.4 mg, 0.2 mmol) was dissolved in 5 ml of methanol at room temperature. The mixture was stirred for one hour. Pale-green single crystals of the title compound suitable for X-ray analysis were obtained by slow evaporation at room temperature for two weeks.

### Refinement

The H atoms bonded to O1W atoms were located in a difference Fourier map and fully refined (positional and isotropic displacement parameters ). Other H atoms were calculated geometrically with C-H distances of 0.93-0.97 Å and were allowed to ride on the C atoms to which they were bonded with *U*_iso_(H) = 1.2*U*_eq_(C).

### Crystal data

**Table:**

[Ni(C_7_H_6_NO_2_)_2_(H_2_O)_2_]	*F*(000) = 380
*M**_r_* = 367.00	*D*_x_ = 1.746 Mg m^−^^3^
Monoclinic, *P*2_1_/*n*	Mo *K*α radiation, λ = 0.71073 Å
Hall symbol: -P 2yn	Cell parameters from 804 reflections
*a* = 8.3346 (12) Å	θ = 3.1–27.8°
*b* = 7.100 (1) Å	µ = 1.43 mm^−^^1^
*c* = 12.1023 (18) Å	*T* = 293 K
β = 102.977 (2)°	Block, pale-green
*V* = 697.87 (17) Å^3^	0.22 × 0.15 × 0.11 mm
*Z* = 2	

### Data collection

**Table:**

Bruker APEXII 1K CCD area-detector diffractometer	1627 independent reflections
Radiation source: fine-focus sealed tube	1471 reflections with *I* > 2σ(*I*)
graphite	*R*_int_ = 0.017
φ and ω scans	θ_max_ = 28.2°, θ_min_ = 2.7°
Absorption correction: multi-scan (*SADABS*; Bruker, 2004)	*h* = −10→11
*T*_min_ = 0.774, *T*_max_ = 0.855	*k* = −8→9
4515 measured reflections	*l* = −16→15

### Refinement

**Table:**

Refinement on *F*^2^	Primary atom site location: structure-invariant direct methods
Least-squares matrix: full	Secondary atom site location: difference Fourier map
*R*[*F*^2^ > 2σ(*F*^2^)] = 0.023	Hydrogen site location: inferred from neighbouring sites
*wR*(*F*^2^) = 0.067	H atoms treated by a mixture of independent and constrained refinement
*S* = 1.00	*w* = 1/[σ^2^(*F*_o_^2^) + (0.0413*P*)^2^ + 0.1727*P*] where *P* = (*F*_o_^2^ + 2*F*_c_^2^)/3
1627 reflections	(Δ/σ)_max_ < 0.001
114 parameters	Δρ_max_ = 0.34 e Å^−^^3^
0 restraints	Δρ_min_ = −0.20 e Å^−^^3^

### Special details

**Table:**

Experimental. Refinement of F^2^ against ALL reflections. The weighted *R*-factor *wR* and goodness of fit S are based on F^2^, conventional *R*-factors *R* are based on F, with F set to zero for negative F^2^. The threshold expression of F^2^ > 2sigma(F^2^) is used only for calculating *R*-factors(gt) *etc*. and is not relevant to the choice of reflections for refinement. *R*-factors based on F^2^ are statistically about twice as large as those based on F, and R– factors based on ALL data will be even larger.
Geometry. All e.s.d.'s (except the e.s.d. in the dihedral angle between two l.s. planes) are estimated using the full covariance matrix. The cell e.s.d.'s are taken into account individually in the estimation of e.s.d.'s in distances, angles and torsion angles; correlations between e.s.d.'s in cell parameters are only used when they are defined by crystal symmetry. An approximate (isotropic) treatment of cell e.s.d.'s is used for estimating e.s.d.'s involving l.s. planes.

### Fractional atomic coordinates and isotropic or equivalent isotropic displacement parameters (Å^2^)

**Table:**

	*x*	*y*	*z*	*U*_iso_*/*U*_eq_	
Ni1	1.0000	1.0000	0.0000	0.01966 (10)	
O1	0.73292 (15)	0.53276 (15)	−0.14497 (10)	0.0320 (3)	
O1W	0.87262 (14)	1.17222 (17)	−0.13481 (9)	0.0315 (2)	
H1WA	0.820 (2)	1.274 (3)	−0.1354 (18)	0.046 (6)*	
H1WB	0.838 (3)	1.115 (4)	−0.199 (2)	0.072 (7)*	
O2	0.89799 (12)	0.77910 (14)	−0.09898 (9)	0.0275 (2)	
N1	0.79922 (16)	0.99744 (14)	0.07548 (10)	0.0221 (3)	
C1	0.70767 (17)	0.8430 (2)	0.08078 (11)	0.0238 (3)	
C2	0.56827 (18)	0.8517 (2)	0.12662 (12)	0.0302 (3)	
H2A	0.5061	0.7438	0.1297	0.036*	
C3	0.5230 (2)	1.0201 (2)	0.16721 (14)	0.0331 (4)	
H3A	0.4307	1.0270	0.1983	0.040*	
C4	0.61676 (17)	1.1788 (2)	0.16100 (13)	0.0313 (3)	
H4A	0.5885	1.2946	0.1871	0.038*	
C5	0.75331 (17)	1.1610 (2)	0.11506 (12)	0.0272 (3)	
H5A	0.8168	1.2676	0.1113	0.033*	
C6	0.76151 (18)	0.6580 (2)	0.04071 (12)	0.0277 (3)	
H6A	0.6757	0.5660	0.0412	0.033*	
H6B	0.8592	0.6165	0.0950	0.033*	
C7	0.79960 (16)	0.65785 (18)	−0.07716 (11)	0.0229 (3)	

### Atomic displacement parameters (Å^2^)

**Table:**

	*U*^11^	*U*^22^	*U*^33^	*U*^12^	*U*^13^	*U*^23^
Ni1	0.02185 (15)	0.01823 (15)	0.01945 (15)	−0.00105 (8)	0.00579 (10)	−0.00173 (8)
O1	0.0420 (7)	0.0236 (5)	0.0281 (6)	−0.0050 (4)	0.0029 (5)	−0.0058 (4)
O1W	0.0397 (6)	0.0257 (6)	0.0263 (6)	0.0069 (5)	0.0012 (5)	0.0001 (4)
O2	0.0320 (5)	0.0251 (5)	0.0268 (5)	−0.0052 (4)	0.0099 (4)	−0.0056 (4)
N1	0.0236 (6)	0.0223 (6)	0.0203 (6)	0.0003 (4)	0.0049 (5)	−0.0012 (4)
C1	0.0261 (7)	0.0270 (8)	0.0173 (6)	−0.0018 (5)	0.0030 (5)	0.0007 (5)
C2	0.0271 (7)	0.0385 (9)	0.0247 (7)	−0.0067 (6)	0.0055 (6)	0.0014 (6)
C3	0.0244 (7)	0.0487 (10)	0.0276 (8)	0.0015 (6)	0.0087 (6)	−0.0019 (6)
C4	0.0297 (7)	0.0367 (8)	0.0274 (7)	0.0063 (6)	0.0064 (6)	−0.0068 (6)
C5	0.0284 (7)	0.0251 (7)	0.0279 (7)	0.0006 (6)	0.0060 (6)	−0.0037 (6)
C6	0.0360 (8)	0.0212 (7)	0.0265 (7)	−0.0050 (6)	0.0082 (6)	0.0008 (5)
C7	0.0255 (6)	0.0179 (7)	0.0240 (6)	0.0035 (5)	0.0028 (5)	−0.0009 (5)

### Geometric parameters (Å, °)

**Table:**

Ni1—O2	2.0397 (10)	C2—C3	1.378 (2)
Ni1—N1	2.0789 (13)	C2—H2A	0.9300
Ni1—O1W	2.1228 (11)	C3—C4	1.383 (2)
O1—C7	1.2515 (17)	C3—H3A	0.9300
O1W—H1WA	0.84 (2)	C4—C5	1.380 (2)
O1W—H1WB	0.87 (3)	C4—H4A	0.9300
O2—C7	1.2572 (17)	C5—H5A	0.9300
N1—C5	1.3444 (17)	C6—C7	1.5294 (19)
N1—C1	1.3454 (17)	C6—H6A	0.9700
C1—C2	1.397 (2)	C6—H6B	0.9700
C1—C6	1.503 (2)		
			
O2^i^—Ni1—N1^i^	88.90 (4)	C1—C2—H2A	120.0
O2^i^—Ni1—N1	91.10 (4)	C2—C3—C4	118.97 (15)
N1^i^—Ni1—N1	180	C2—C3—H3A	120.5
O2^i^—Ni1—O1W	94.53 (4)	C4—C3—H3A	120.5
N1—Ni1—O1W	91.70 (5)	C5—C4—C3	118.35 (14)
O2^i^—Ni1—O1W^i^	85.47 (5)	C5—C4—H4A	120.8
N1—Ni1—O1W^i^	88.30 (5)	C3—C4—H4A	120.8
O1W—Ni1—O1W^i^	180	N1—C5—C4	123.29 (14)
Ni1—O1W—H1WA	132.0 (14)	N1—C5—H5A	118.4
Ni1—O1W—H1WB	115.2 (17)	C4—C5—H5A	118.4
H1WA—O1W—H1WB	109 (2)	C1—C6—C7	116.18 (11)
C5—N1—C1	118.50 (13)	C1—C6—H6A	108.2
C5—N1—Ni1	118.07 (9)	C7—C6—H6A	108.2
C1—N1—Ni1	123.31 (9)	C1—C6—H6B	108.2
N1—C1—C2	120.96 (13)	C7—C6—H6B	108.2
N1—C1—C6	118.88 (12)	H6A—C6—H6B	107.4
C2—C1—C6	120.11 (13)	O1—C7—O2	124.25 (13)
C3—C2—C1	119.92 (14)	O1—C7—C6	117.23 (12)
C3—C2—H2A	120.0	O2—C7—C6	118.50 (12)

Symmetry codes: (i) −*x*+2, −*y*+2, −*z*.

### Hydrogen-bond geometry (Å, °)

**Table:**

*D*—H···*A*	*D*—H	H···*A*	*D*···*A*	*D*—H···*A*
O1W—H1WA···O1^ii^	0.84 (2)	1.97 (2)	2.8035 (17)	169.7 (19)
O1W—H1WB···O1^iii^	0.87 (3)	1.93 (3)	2.7936 (17)	169 (2)

Symmetry codes: (ii) *x*, *y*+1, *z*; (iii) −*x*+3/2, *y*+1/2, −*z*−1/2.

## References

[bb1] Bruker (2004). *APEX2*, *SAINT* and *SADABS* Bruker AXS Inc, Madison, Wisconsin, USA.

[bb2] Faure, R. & Loiseleur, H. (1972). *Acta Cryst.* B**28**, 811–815.

[bb3] Faure, R. & Loiseleur, H. (1975). *Acta Cryst.* B**31**, 1472–1475.

[bb4] Sheldrick, G. M. (2008). *Acta Cryst.* A**64**, 112–122. 10.1107/S0108767307043930 18156677

